# Zingiberis Rhizoma Recens: A Review of Its Traditional Uses, Phytochemistry, Pharmacology, and Toxicology

**DOI:** 10.1155/2021/6668990

**Published:** 2021-03-02

**Authors:** Xing Li, Mingyue Ao, Chunling Zhang, Shunming Fan, Zhimin Chen, Lingying Yu

**Affiliations:** College of Pharmacy, Chengdu University of Traditional Chinese Medicine, Chengdu 6111137, China

## Abstract

Zingiberis Rhizoma Recens (ZRR, the fresh rhizoma of *Zingiber officinale* Roscoe) is a widely used traditional Chinese medicine (TCM). It is also a traditional spice, widely used around the world. The present paper reviews advances in research relating to the botany, ethnopharmacology, phytochemistry, pharmacology, and toxicology of Zingiberis Rhizoma Recens. In addition, this review also discusses some significant issues and the potential direction of future research on Zingiberis Rhizoma Recens. More than 100 chemical compounds have been isolated from Zingiberis Rhizoma Recens, including gingerols, essential oils, diarylheptanoids, and other compounds. Modern studies have confirmed that Zingiberis Rhizoma Recens has pharmacological effects on the nervous system and cardiovascular and cerebrovascular systems, as well as antiemetic, antibacterial, antitumor, anti-inflammatory, and antioxidant effects. However, the modern studies of Zingiberis Rhizoma Recens are still not complete and more bioactive components and potential pharmacological effects need to be explored in the future. There is no unified standard to evaluate the quality and clinical efficacy of Zingiberis Rhizoma Recens. Therefore, we should establish reasonable, accurate, and reliable quality control standards to make better use of Zingiberis Rhizoma Recens.

## 1. Introduction

Zingiberis Rhizoma Recens (ZRR) is the fresh rhizoma of *Zingiber officinale* Roscoe and has a long history as an effective traditional Chinese medicine. The use of ZRR can be traced from the *Shen Nong Ben Cao Jing.* In Chinese Pharmacopoeia, ZRR has the function of relieving exterior syndrome and dispersing cold and warming middle energizer to arrest vomiting. It is mostly used to treat cold, vomiting, and cough caused by wind weather. ZRR is distributed in China, India, Indonesia, Nigeria, and other countries and is famous for its antiemetic effect.

In recent years, many researchers have carried out in-depth research on the phytochemical and pharmacological effects of ZRR and made significant progress. Modern phytochemical studies have shown that the main components of ZRR include gingerols, essential oils, diarylheptanoids, and other compounds. Pharmacological studies have also shown that ZRR has a wide range of pharmacological effects, including effects on the nervous system and cardiovascular and cerebrovascular systems, as well as antiemetic, antibacterial, antitumor, anti-inflammatory, and antioxidant effects. This review provides information on the botanical characterizations, traditional usages, chemical constituents, pharmacological activities, toxicity, and quality control of ZRR. In this paper, we hope to have a comprehensive understanding of the effects of ZRR and also provide a basis for further research.

## 2. Botany

**Figure 1:**
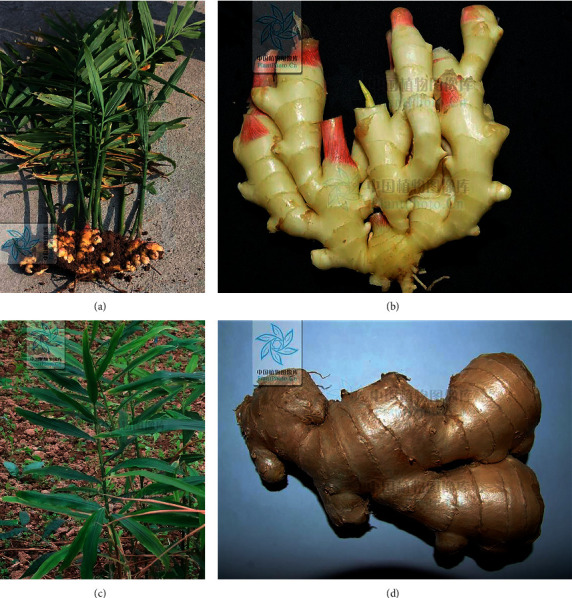
The entire *Z*. *officinale* plant (a), the underground part of *Z*. *officinale* (b), the aboveground part of *Z*. *officinale* (c), and ZRR (the fresh rhizoma of *Z. officinale*) (d) (cited from Flora Republicae Populairs Sinicae at http://ppbc.iplant.cn).

ZRR is known as “Shengjiang” in China and is the fresh rhizoma of *Zingiber officinale* Roscoe (*Z. officinale*) (Figure 1), and the plant is widely cultivated in central, southeast, and southwest of China.

As far as the growing environment is concerned, *Z. officinale* is generally distributed in the tropical and subtropical regions and is widely cultivated in many countries of southern and eastern of Asia including India, Indonesia, Nigeria, and Thailand.

*Z. officinale*, a member of the Zingiberaceae family, likes a warm and humid climate. Its cold resistance and drought resistance are weak, and its plants can only grow in the frost-free period. The optimum temperature for growth is 25–28°C. It primarily thrives in moist and nonwater sandy loam that is loose and fertile. The plant is approximately 0.5–1 m tall and has fleshy, branched rhizomes. This plant is aromatic and spicy. The leaves are lanceolate or linear-lanceolate, its apex is acuminate, and its base is attenuated to narrow, glabrous, and sessile. The inflorescence is ovoid, the bracts are ovate, pale green, or yellowish at margin, and its apex is mucronate. The calyx tube is about 1 cm long; the flower is yellowish green, and the lobes are lanceolate. The flowering period is autumn [1]. ZRR is harvested in autumn and winter, the fibrous roots are removed, and then rhizoma is used for medicinal purposes.

## 3. Ethnopharmacology

### 3.1. Traditional Use

ZRR is a kind of Chinese herbal medicine with a wide range of pharmacological effects and plays an indispensable role in the healthcare industry in China for a long time.

In the Chinese Pharmacopoeia (version 2020), this herb is described as being slight warm in property and acrid in flavour. It affects the spleen, stomach, and lung and acts to relieve exterior syndrome and disperse cold, warm middle energizer to arrest vomiting, dissipate phlegm and relieve cough, and resolve the toxin of fish and crabs; therefore, it can be applied in the treatment of common cold due to wind-cold, vomiting due to stomach cold, cough resulting from cold phlegm, and poison from fish and crabs. The recommended dosage is 3–10 g. Because ZRR has a strong effect on promoting fire in the body and may cause damage to Yang Qi, individuals with excessive heat or yin deficiency and internal heat should avoid to use it [2].

In China, the use of ZRR can be traced from the spring and autumn periods (770-476 BC), or even earlier. It was mentioned in the *Shen Nong Ben Cao Jing* and was classified as a middle product. Middle product usually refers to nontoxic or very small toxicity, which has the effect of tonifying deficiency and strengthening weakness or eliminating pathogenic factors and resisting diseases. It is mainly used to adjust the body, but it cannot be taken for a long time [3].

ZRR has a wide range of effects. It is often used in combination with other Chinese medicines but is generally not used alone. In the major prescription books in ancient China, *Shang Han Za Bing Lun*, there are 71 prescriptions containing ZRR, including 38 in the Treatise on *Shang Han Lun* and 33 in the Outline of *Jin Gui Yao Lue*. The parts of formulations are shown in Table 1.

### 3.2. Processing

Before Chinese herbal medicines are used, they are usually processed by the methods of cleansing, cutting, heating, or combining with processed excipients. The purpose of these processes is to improve the efficacy or reduce the toxicity of the product.

*Jin Gui Yao Lue* recorded the method of cutting in the Han dynasty. According to *Lei Gong Pao Zhi Lun*, there was a method of peeling in the Northern and Southern dynasties. *Lishang Xuduan Fang* recorded that there was the method of roasting in the Tang dynasty. The Ming dynasty had the stir-fry (*Jing Yue Quan Shu*), the sun-drying method (*Ben Cao Meng Quan*), and so on. In the current version of the Chinese Pharmacopoeia, only two processing methods have been reported: sun-drying or low-temperature drying.

However, processing has an unavoidable impact on the composition and efficacy of medicinal materials. We also reviewed the content change studies of ZRR before and after processing and found that the content of volatile oil decreased and the content of gingerols and polysaccharides increased [6–8]. The changes of these components are mainly quantitative, and the deep-seated relationship between them and the changes of pharmacodynamics require further study. Therefore, different processed products have different clinical effects and appropriate processed products should be selected according to the condition.

## 4. Phytochemistry

A literature search identified over 100 chemical constituents that have been isolated from *Z. officinale*. The main constituents are gingerols, essential oils, diarylheptanoids, and others. The compounds isolated are listed in Tables 2–4, and their corresponding structures are also presented.

### 4.1. Gingerols

Gingerol is the general term of the pungent ingredients in *Z. officinale*, which is a mixture of many substances. Gingerols are not only the main components of characteristic spicy flavor, but also the main functional components of various biological activities. The structure of gingerols contains the 3-methoxy-4-hydroxyphenyl functional group. According to the different fatty chains connected to this functional group, it can be divided into the following types:

#### 4.1.1. Gingerols

**Figure 2:**
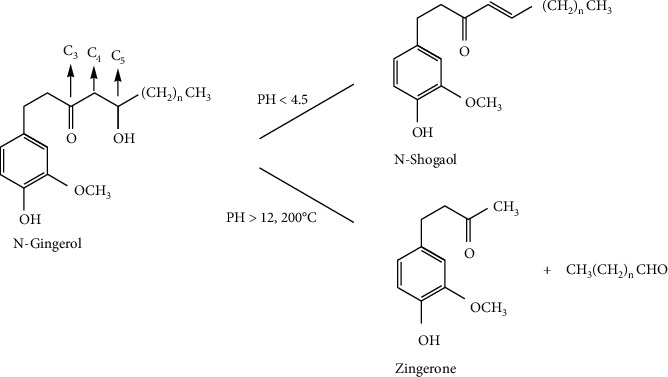
Gingerols and its chemical changes [11].

At present, there are more than ten clear gingerols, including 6-, 8-, 10-, and 12-gingerol. The properties and structures of these components are similar. There are C_3_ carbonyl and C_5_ hydroxy (i.e., *ß*-hydroxyketone structure) in their molecular structures, which makes the chemical properties of gingerols extremely unstable. For example, in acid condition (pH < 4.5), the active hydrogen of C_4_ is easy to dehydrate with the hydroxyl of C_5_ to form shogaols; in heating or alkaline condition, the carbon-carbon bond between C_4_ and C_5_ breaks to form zingerone and corresponding aldehydes. The transformation of gingerols under different conditions is shown in Figure 2. In addition, the length of the side chain, the acetoxy group at the 3, 5 position, the methoxy group on the aromatic ring, and the phenolic hydroxyl functional group all affect the biological activity of gingerols [11].

#### 4.1.2. Shogaols

It is considered that the content of shogaols in *Z. officinale* is very small, which is formed by dehydration of gingerols during storage and processing. The content of shogaols in *Z. officinale* is 6-shogaol > 10-shogaol > 8-shogaol > 4-shogaol [21].

#### 4.1.3. Others

**Figure 3:**
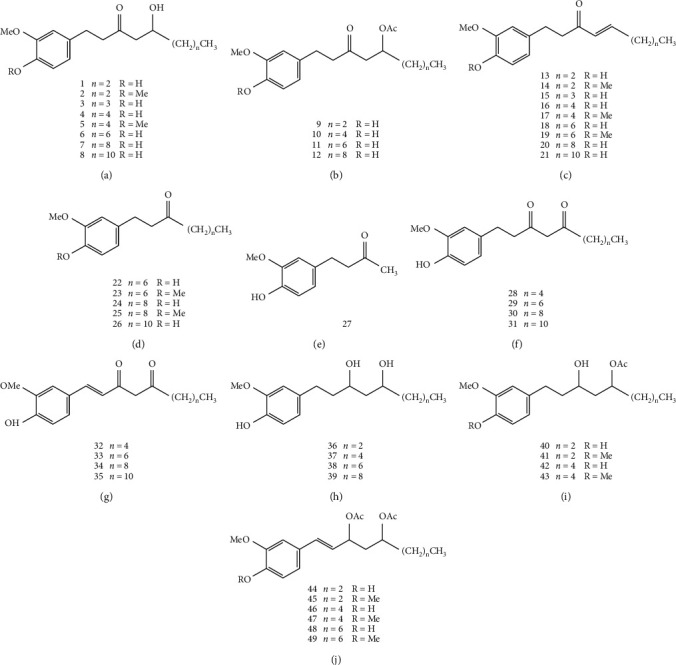
The structures of gingerols from *Z. officinale*.

In addition, gingerols also contain other components, such as zingerone, paradols, gingerdiones, and gingerdiols. The part of the structural formula is shown in Figure 3.

### 4.2. Essential Oils

**Figure 4:**
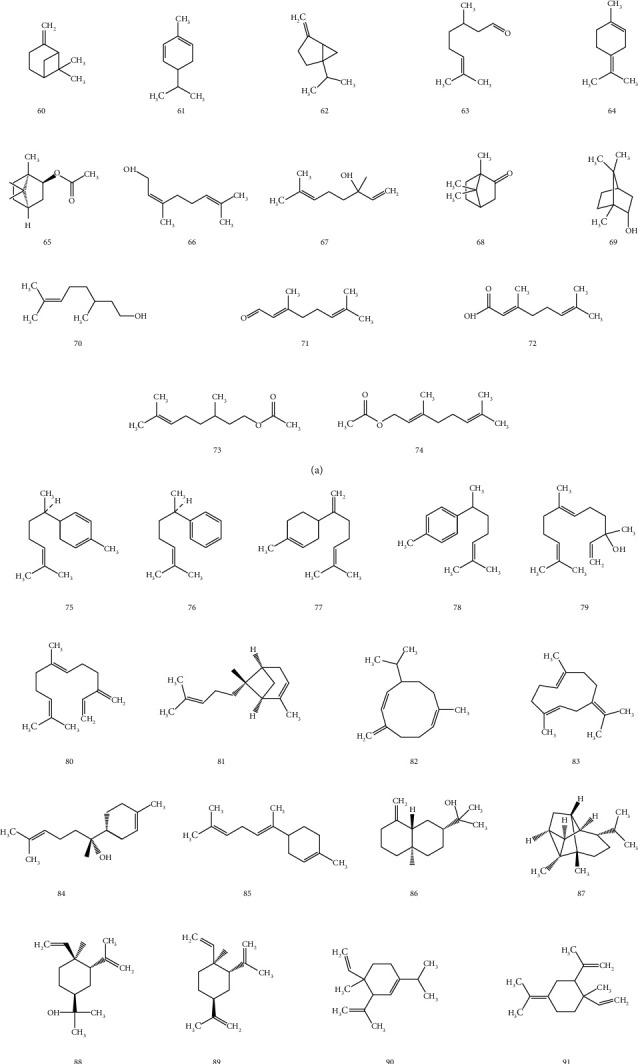
The structures of essential oils from *Z. officinale*: (a) monoterpenoids; (b) sesquiterpenoids.

Essential oil is also one of the main components of *Z. officinale* and is mainly composed of pinene, phellandrene, and camphene [22]. The total content of essential oil is 0.25%–3.0%. The essential oil component is dominated by terpenoids and is mainly composed of sesquiterpene carbohydrate (50%–66%) and sesquiterpene oxide (17%). The part of structural formula is shown in Figure 4.

### 4.3. Diarylheptanoids

**Figure 5:**
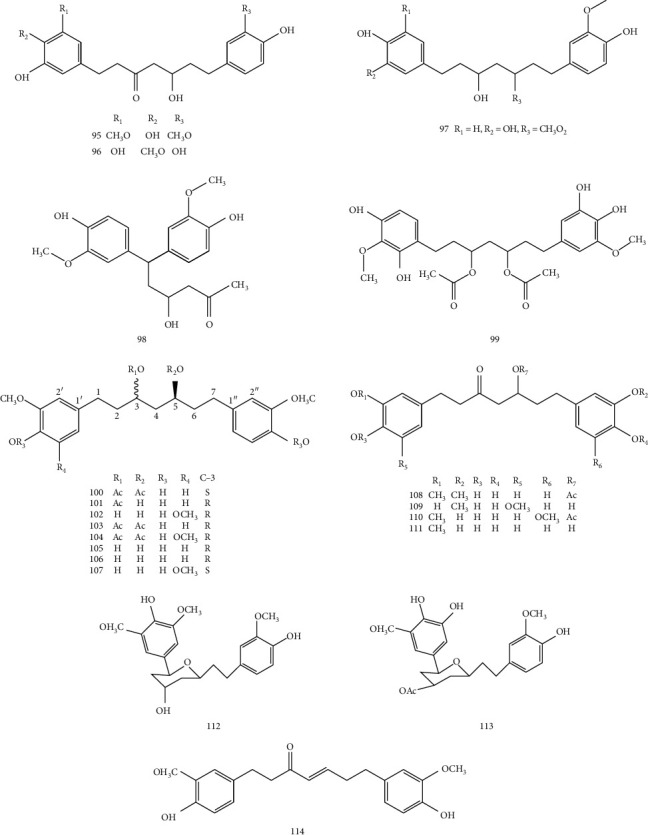
The structures of diarylheptanoids from *Z. officinale*.

Diarylheptanoids are a group of substances with nuclear structure of 1, 7-diarylheptanoid. According to their structural characteristics, they can be divided into two groups: cyclic diarylheptanoid and linear diarylheptanoid. The chemical structure of linear diarylheptanoid is characterized by the position of two phenyl substituents at 1, 7 of heptane skeleton, the substituents on phenyl are methoxy or hydroxy, and the substituents are in para- or interposition; there are 3 types of substituted diphenyl groups: phenyl, 4-hydroxyphenyl, and 3-methoxy-4-hydroxyphenyl [23]. The structural formula is shown in Figure 5.

### 4.4. Other Components

Besides the aforementioned, phenylpropanoids, flavonoids, nucleosides, polysaccharides, proteins, cellulose, and a large number of carbohydrates and trace elements have been isolated from *Z. officinale* [9]. Zhang [24] found that it contained mineral elements (including Fe, Al, Mn, Na, and Zn) and 17 amino acids, such as Asp, Glu, Arg, Leu, and Gly.

## 5. Pharmacological Activities

In recent years, many researchers have conducted in-depth research on the pharmacological effects of extracts or isolated compounds from ZRR and have found that ZRR has a wide range of pharmacological effects (Table 5). Modern pharmacological studies confirmed the traditional efficacy of ZRR, particularly in antiemetic. The main mechanism of action may be related to the 5-HT receptor, but further studies are required to define the related mechanisms. Pharmacological studies in animals experiments also showed that ZRR has potential as a treatment for Alzheimer's disease (AD) and cognition. The antioxidant and anti-inflammatory effects of ZE and its isolated compounds also indicated pharmacological effects in cardiovascular and cerebrovascular fields. In addition, in vitro and in vivo pharmacological studies have shown that ZE and compounds have antibacterial and antitumor effects.

We will introduce the research progress of modern pharmacology of ZRR from its effects on the nervous system and cardiovascular and cerebrovascular systems and will discuss reported antiemetic, antibacterial, antitumour, anti-inflammatory, and antioxidative effects.

### 5.1. Pharmacological Effects on the Nervous System

Modern pharmacological studies have shown that ZRR can be used to treat nervous system diseases, such as AD, and has the effect of improving cognitive function. Although the mechanisms of these effects have been studied, to better develop the medicinal value of ZRR, the related mechanisms of action require more study.

#### 5.1.1. Treatment of Alzheimer's Disease

Experimental evidence suggested that learning and memory impairment in mouse could be improved by 6-shogaol both in vitro and in vivo. 6-Shogaol decreased the levels of CysLT1R/cathepsin B and amyloid-beta in the brain of mice. Therefore, 6-shogaol may be beneficial for treating AD due to its role as a CysLT1R and cathepsin B inhibitor [34]. Further research showed that 6-gingerol activated Akt activity and inhibited GSK-3*β* activity, thereby protecting PC12 cells against *Aβ*_1−42_-induced apoptosis through the PI3K/Akt/GSK-3*β* signaling pathway, suggesting that 6-gingerol may be one of the effective intervention measures for AD [35].

Another researcher investigated whether fermented ginger (FG) had a neuroprotective effect in an AD mouse model. These researchers found that FG alleviated memory dysfunction induced by the *Aβ*_1−42_ plaque toxicity and inhibited *Aβ*_1−42_ plaque-induced neuronal cell loss in the CA3 region of the hippocampus. Thus, these studies showed the antiamnesic effects of FG against the *Aβ*_1−42_ plaque toxicity by inhibiting neuronal cell loss and synaptic disturbance, besides suggesting that FG can alleviate AD-like memory dysfunction and neuronal degradation [36].

#### 5.1.2. Improving Cognition

Research using an improved Morris water maze test revealed that ZRR (4 g/kg) reduced the impairment of lead-induced learning and memory in rats [37]. Zhang et al. studied whether 6-gingerol could reduce memory dysfunction and neuroinflammation in rats by administration of lipopolysaccharide (LPS). The researchers found that 6-gingerol (2 mg/kg) increased cognitive ability and inhibited the over activation of astrocytes. Therefore, the study indicated that 6-gingerol played an effective neuroprotective role, most likely through its antioxidant and anti-inflammatory activities [38].

### 5.2. Pharmacological Effects on Cardiovascular and Cerebrovascular Systems

Treatment of cardiovascular and cerebrovascular systems is not one of the traditional uses of ZRR, but they have been identified as potential targets as part of an in-depth study of the pharmacological effects of ZRR. Moreover, with the development of modern separation and purification technology, the compounds in ZRR also have protective effects on the cardiovascular and cerebrovascular systems, such as 6-gingerol. The results showed that its mechanisms are closely related to antioxidant and anti-inflammatory effects.

#### 5.2.1. Cardioprotective Activity

The research conducted by El-Hawwary et al. was aimed at further evaluating the mechanism by which ZRR protected cisplatin-induced myocardial injury. They believed that ZRR protected the cardiotoxicity caused by cisplatin through downregulating the TNF-*α* and P53 immune expression and reducing the levels of LDH and CK [39].

Further research showed that 6-gingerol reduced the content of serum malondialdehyde and increased superoxide dismutase content. By inhibiting oxidative stress, 6-gingerol alleviated the damage of cardiac myocytes, indicating that 6-gingerol had cardioprotective effect [40]. 6-Gingerol inhibited the apoptosis of AC16 cardiomyocytes induced by ischemia-reperfusion and reduced the level of ROS [41]. The cardioprotective activity of 6-gingerol may be mediated by inhibiting L-type Ca^2+^ in rat cardiomyocytes and reducing extracellular Ca^2+^ influx to reduce intracellular Ca^2+^ [42].

Investigation of the recovery of regional myocardial function in thoracotomy before a 30-minute left anterior descending coronary artery ligation, given pretreatment with 6-gingerol at a dose of 6 mg/kg (intravenous injection), 6-gingerol pretreatment decreased the level of markers of myocardial injury, which demonstrated that 6-gingerol protected myocardium to a certain extent [43].

When Ren et al. studied the protective mechanism of 6-gingerol on cardiomyocytes H9c2 cells, they found that 6-gingerol had cytoprotective effect on hypoxia-induced injury in a concentration-dependent manner at a lower concentration (5–50 *μ*m). Their experimental results showed that 6-gingerol inhibited the expression of BNIP3 and activated the PI3K/AKT/mTOR signaling pathway, which resulted in the inhibition of apoptosis and autophagy [44]. Whether there are other mechanisms of cardioprotective activity of ZRR requires further study.

#### 5.2.2. Cerebral Ischaemia Protection

Studies have found that ZRR has protective effects against focal cerebral ischemia-reperfusion (FCI-R) injury. For example, total phenol of ZRR was reported to improve the neurological symptoms of MCAO rats, prolonged the passive conditioned reflex latency and reduced the number of errors, and significantly reduced the area of cerebral infarction. Brain histopathological examination revealed that the number of bleeding cases and neutrophils in the treated group were significantly lower than untreated group [45]. Jia et al. showed that the protective effect of ZAE on FCI-R injury could be attributed to apoptosis signal relative protein. ZAE significantly reduced caspase-3 gene expression, decreased Bax and Bcl-2 positive cells, and increased the ratio of Bcl-2/Bax [46].

In addition to FCI-R injury, ZRR also had a protective effect on cerebral ischemia-reperfusion (CI-R) injury. The experimental results of Wang et al. confirmed the protective effect of ZAE (100 mg/kg) on CI-R injury, which seems to be associated with inflammatory factors and adhesion factors, involving changes in the in vivo activity of TNF-*α*, ICAM-1, and P-selectin [47]. ZAE (0.2 g/kg) significantly mitigated brain tissue edema in rats, prolonged thrombin time, and reduced plasma fibrinogen content. It suggested that protective effect of ZAE is partially related to its anticoagulant effect [48].

In the treatment of CI-R, polysaccharide of ZRR enhanced the SOD activity of brain tissue to inhibit the production of free radicals, promote lipid peroxidation, and reduce the production of MDA. In addition, polysaccharide of ZRR has been shown to reduce the content of NO in brain tissue [49].

### 5.3. Antiemetic Effect

ZRR is a traditional Chinese medicine (TCM) widely used to relieve vomiting in Asian countries. Many experts and scholars have studied the antiemetic effect of ZRR. The antiemetic effect of ZRR may be related to the 5-HT_3_ receptor, M_3_ cholinocepter in gastrointestinal tract [50], and NK_1_ receptor [51]. For example, Walstab et al. found that ZSE inhibited Ca^2+^ influx of 5-HT_3_ receptor, and noncompetitively inhibited the activation of human 5-HT_3_ receptor [52]. Some scholars have studied the effects of ZAE (600, 300, and 150 mg/kg) on vomiting in rats and found that ZAE had antiemetic effect, and its mechanism may be related to substance P, 5-HT, and the vomiting center in the central nervous system, which provided a basis for investigations into the use of the ZRR as a drug [53].

Some scholars have also studied the pharmacological effects of different processed products of ZRR on vomiting. Ginger juice (4 g/kg, 2 g/kg) and ginger decoction (10 g/kg, 5 g/kg) could reduce the amount of kaolin intake, reflecting ginger had antiemetic effect. The ginger juice low-dose group (2 g/kg) had the best antiemetic effect, and its antiemetic substance may be 6-gingerol [54]. In an experiment comparing the effect of dried ginger juice (DGJ), fresh ginger juice (FGJ), and fresh ginger boiled juice (FGBJ) in relieving the emetic effects, the study found that the above ginger juice groups reduced the intake of kaolin compared with the cisplatin group, indicating that all kinds of ginger juice could be effective prevent vomiting [10].

In addition to the above mentioned pharmacological effects, research has been conducted into the medicinal effects of ingredients isolated from ZRR, such as 6-gingerol and 6-shogaol. For example, in a study about the effects of 6-gingerol and 6-shogaol on isolated guinea pig ileum contraction, the study found that the mechanism of antiemetic may be noncompetitive blocking of the 5-HT_3_ receptor by 6-gingerol and 6-shogaol [29]. 6-Gingerol and 6-shogaol could also noncompetitively block the NK_1_ receptor, which suggested that 6-gingerol and 6-shogaol may be the main active ingredients of ZRR for alleviating vomiting [51].

ZRR has been widely used for nausea and vomiting since ancient times, and modern research has also confirmed the antiemetic effect of ZRR during pregnancy, chemotherapy, and surgery. For pregnant women with mild to moderate nausea and vomiting in the early stage of pregnancy, ZRR (500 mg) and vitamin B6 (40 mg) were administered twice a day, and the effect is equivalent. However, ZRR is more effective for severity of nausea and amount of vomiting [55].

In an experiment, forty-seven patients with gynecological malignancies were enrolled for chemotherapy with carboplatin and paclitaxel. The results showed that ZRR significantly reduced the nausea and vomiting of the patients. However, the results of delayed and acute phase were still unclear [56].

Chang et. concluded oral administration of 0.5–2.0 g/d ZE mitigated acute CINV in patients, especially acute vomiting [57]. Konmun et al. found that a major bioactive constituent of ZRR, 6-gingerol, exerted antiemetic effects in patients who received moderately to highly emetogenic adjuvant chemotherapy [58]. In one experiment, 100 patients with cholelithiasis who were candidated for laparoscopic cholecystectomy were treated using TCM Zingiberis Rhizoma Recens (500 mg) as the treated group, using the Western medicine ondansetron (4 mg) as a control. The results showed that the incidence of postoperative nausea in the treated group was significantly lower than the ondansetron group, indicating that the treatment of PONV with ZRR improved symptom to a certain extent [59].

### 5.4. Antimicrobial Effect

Many in vitro antibacterial studies have shown that ZRR had broad-spectrum antibacterial effects. The volatile oil from ZSE had an antibacterial effect on many kinds of bacteria, not only on the Gram-positive bacteria such as *Staphylococcus aureus* and *Bacillus subtilis*, but also on the Gram-negative bacteria such as *Escherichia coli* and *Salmonella.* In addition, volatile oil from ZRR had a stronger inhibiting ability to fungus compared with bacteria [60].

ZEE has been reported to have antimicrobial effects against *Pseudomonas aeruginosa*, *Escherichia coli*, *Staphylococcus aureus*, *Klebsiella pneumoniae*, *Bacillus cereus*, *Acinetobacter baumannii*, *Candida albicans*, *and Candida krusei.* The MICs of ZRR against the above bacteria are 40, 40, 20, 20, 20, 20, and 10, 5 mg/mL, respectively [61]. In broth microdilution assays, ZME exhibited antibacterial activities against *Klebsiella pneumoniae* (MIC = 12.5 *μ*g/mL). With the increase of extract concentration, the inhibition zone increased. When the concentration of the extract was 100 *μ*g/mL, *Klebsiella pneumonia* showed the highest susceptibility (29.04 ± 0.35 mm), followed by *Pseudomonas aeruginosa*, *Escherichia coli*, and *Salmonella typhi*, while *Staphylococcus aureus* had the least inhibition zone [33]. Different concentration of ZEE had different degrees of antibacterial effect, and the most antibacterial effect was at 75% ethanol solution. And the effect was as follows: *Bacillus subtilis* > *Yeast* > *Staphylococcus albus* > *Escherichia coli* *>* mixed microbes from egg surface [62].

Furthermore, essential oils, polyphenols, flavonoids, and gingerols in ZRR also had antimicrobial effects. The resazurin microtiter assay plate and broth microdilution method were used to investigate the antibacterial effect of ZRR in vitro. It was found that volatile oil from ZRR inhibited *Mycobacterium tuberculosis* and *Nontuberculous mycobacteria* [14]. The inhibition rate of polyphenol from ZRR to *Escherichia coli* was 75% [63]. Flavonoids from ZRR had different degrees of inhibitory effect on the five kinds of bacteria, and the order of the inhibitory effect was *Bacillus subtilis* > *Aspergillus niger* > *Staphylococcus aureus* > *Penicillium* > *Escherichia coli* [64]. In the range of test concentration, gingerols had antibacterial activity against Gram-negative and Gram-positive bacteria, but the antibacterial effect is moderately sensitive (10–15 mm), and the antibacterial activity decreased with the decrease of gingerol concentration [65].

### 5.5. Antitumour Effect

ZAE (100 mg/kg) significantly inhibited gastric cancer in *N*-nitroso *N*-methyl urea (MNU)-induced albino Wistar rats. The inhibitory effect was associated with combating pathological changes such as inflammation and oxidative stress [66]. ZEE inhibited the growth and proliferation of TM_4_ cell (Sertoli cells of normal mouse testis). As the treatment concentration increased, this inhibitory effect became stronger and stronger in a dose-dependent manner. When the treatment concentration reached 100 *μ*g/mL, the growth and proliferation of TM_4_ cells had been basically inhibited [67]. The crude flavonoid of ZE inhibited the proliferation of the hepatocellular carcinoma cell line HepG2 in a time and dose-dependent manner and induced HepG2 cell apoptosis through a mitochondria-mediated apoptosis pathway [68].

As chemical extraction and separation technology progresses, research on the anticancer effects of ZRR has been concentrated in several major components of ZRR, such as 6-gingerol, 6-shogaol, and other ingredients.

According to reports in the past years, 6-gingerol has been proven to have potential anticancer activities against renal-cell carcinoma [69], human oral (SCC4, KB) and cervical cancer (HeLa) cell lines [70], human colon cancer cell LoVo [71], human breast cancer MCF-7 and MDA-MB-231 [72], human umbilical vein endothelial cells [73], osteosarcoma cells [74], and human gastric adenocarcinoma cell line [75]. Its mechanisms included inhibiting the growth of tumour cells, inducing cell cycle arrest and inducing apoptosis. The antitumor effect of 6-gingerol on renal cancer cells in vivo and in vitro showed that 6-gingerol inhibited the growth of ACHN, 786-O, and 769-P cells in a time- and dose-dependent manner mainly through the AKT-GSK 3*β*-cyclin D1 signaling pathway to induce G1 cell cycle arrest and decrease Ki-67 expression in the nucleus [69]. Additionally, 6-gingerol exerted an antitumor effect by inducing cell apoptosis and inducing cell cycle arrest, including G2 phase arrest of KB and HeLa cells, and S phase arrest of SCC4 cells [70].

6-Shogaol has been demonstrated to have potential anticancer activities towards human renal-cell carcinoma 786-O [76] and ovarian cancer cell lines A2780 [77], and head and neck squamous cell carcinoma cell lines [78]. Studies showed that 6-shogaol had a dose-dependent antiproliferative effect and induced the apoptosis of head and neck squamous cell carcinoma cell lines by downregulating survivin [78]. 6-Gingerol inhibited STAT-3 translocation of ovarian cancer cells to inhibit cell proliferation and induce apoptosis [77].

Additionally, zingerone, one of the major active phenolic agents of ZRR, significantly reduced cell viability, improved the production of reactive oxygen species, and induced apoptosis of breast cancer cell line MCF-7 [79]. Zingerone reduced the expression of cyclin D1, induced the cleavage of caspase-3 and PARP-1, which arrested mitosis, thereby inhibiting the growth of human neuroblastoma cells [80]. Yet, the anticancer effects of ZRR have been studied insufficiently, and more pharmacological and mechanistic research is required.

### 5.6. Anti-Inflammatory Effect

ZME showed potential antiarthritis effects in membrane stabilization assay, proteinase inhibitory assay, and protein denaturation inhibition assays. The inhibition rates in the above three experiments were 84.72 ± 1.38%, 82.72 ± 1.48%, and 81.68 ± 1.66%, respectively [81]. When Ezzat et al. investigated the anti-inflammatory effect of ZAE on carrageenan-induced rat paw oedema in rats, they found that ZAE exerted an anti-inflammatory property by reducing PGE2 level and improving inflammatory markers [82].

Several constituents isolated from ZRR have been demonstrated to ameliorate a number of inflammatory responses. 6-Shogaol appeared to alleviate the lung inflammation of mice induced by house dust mite antigen in vivo [83]. 6-Gingerol prevented chronic ulcerative colitis through downregulating NF-kB (p65) and suppressing proinflammatory cytokines [84]. In addition to chronic colitis, 6-gingerol ameliorated acute colitis by activating adenosine adenosine monophosphate-activated protein kinase [85].

When exploring the anti-inflammatory mechanism of ZRR and its active ingredients, it was found that 6-shogaol (20 *μ*g) inhibited ultraviolet radiation B-induced inflammatory response, including reducing the expression of COX-2 and iNOS and reducing the expression of IL-6, IL-10, and TNF-*α* in human epidermal keratinocytes [86]. A study of 6-shogaol showed that it inhibited the inflammatory response of the ligated periodontitis model in mice, and 6-shogaol attenuated the inflammatory response by reducing the number of macrophages and neutrophils in periodontal tissues and reducing the expression of IL-1*β* and TNF-*α* and, therefore, may be a therapeutic agent against periodontitis [87]. Given that 6-gingerol reduced PGE2 levels and inhibited inflammation-related osteoclast differentiation, 6-gingerol could be used as a potential treatment for inflammatory bone loss [88].

In summary, the extracts and isolates of ZRR have anti-inflammatory effects. Furthermore, on the basis of defined mechanisms and safety profiles, ZRR has the potential to be developed as new anti-inflammatory drugs.

### 5.7. Antioxidative Effect

In DPPH free radical scavenging activity, *ß*-carotene bleaching test, ferric reducing power determination assay, and ZME showed stronger antioxidant activity than ZAE. In addition, the antioxidant activity of ZE was evaluated by the determination of thiobarbituric acid reactive substances and malondialdehyde dosage [89].

Some pharmacological effects of ZRR are achieved through antioxidant action. For example, Mohammed conducted experiments to evaluate whether ZEE effectively protect the thyroid oxidative damage induced by bisphenol A in male rats. The authors proposed that ZEE protected the thyroid oxidative damage and thyroid hormone disorder by activating Nrf2/HO-1 gene expression and promoting the synthesis of thyroxine [90]. In a model of chronic ulcerative colitis in mice induced by dextran sodium sulfate, 6-gingerol effectively protected the colon from oxidative damage by enhancing the antioxidant capacity of cells and inhibiting lipid peroxidation [84]. The protective effect of ZE on human chondrocytes induced by IL-1*β* could be due to its antioxidant properties to lead to a reduction in oxidative stress [91]. Ginger oleoresin (10^−4^ g/mL) reduced ionizing radiation-induced damage to human mesenchymal stem cells by reducing ROS production and altering nuclear translocation of Nrf2 [92]. Therefore, the antioxidant effect of Zingiberis Rhizoma Recens is not a single pharmacological effect but is closely related to other effects and is one of the possible mechanisms for the realisation of other pharmacological effects.

## 6. Toxicology

Modern toxicology studies have shown that ZRR has no oral toxicity and no genotoxicity. Part of the experimental results and data of the ZRR are listed in Table 6.

For ZRR, the toxicity of various extracts was evaluated. The acute toxicity test showed that the methanol extract and the aqueous extract (2 times/day) were fed for 7 days, and no abnormal changes were observed, and all the mice survived. Compared with male mice, the recovery of female mice was slower [93]. The oral toxicity evaluation showed that after increasing doses of ZE (2, 4, 6, 8 and 10 g/kg) which were given to mice for 14 days, no toxic effects were observed compared with distilled water [89].

Although daily clinical administration of ZRR usually does not cause any significant adverse effects in humans, further clinical trials are required to better assess the safety of ZRR.

## 7. Quality Control

The quality of ZRR is affected by different factors such as growing environment and different producing areas. In addition, harvest time, processing methods, and storage conditions affect the quality of ZRR. The medicinal part of ZRR is the rhizoma, and it is often tangled whether to peel it. Due to these factors, the content of active ingredients in ZRR is different, and the quality and clinical efficacy of ZRR cannot be controlled at present.

In the current Pharmacopoeia of various countries, the quality control of *Z. officinale* is mainly carried out through qualitative identification by thin layer chromatography (TLC) and content is determined by high-performance liquid chromatography (HPLC).  Table 7 shows the contents of *Z. officinale* in Pharmacopoeia of various countries.

In the Chinese Pharmacopoeia, TLC and microscopic observation are mainly used for qualitative identification and determination of content by HPLC, and gingerol detection is mainly used to evaluate the quality of ZRR. 6-Ginerol, 8-gingerol, and 10-gingerol are chosen as three marker components to control the quality of ZRR, and it is required that the content of these marker components should be no less than the content specified in the method. ZRR and its processed products are often used in clinical practice.

TCM has characteristics of multicomponent and multitarget. Hence, it is insufficient to evaluate the quality of TCM in terms of the contents of a few compounds. Yet, chemical models of multicomponent control are increasingly common in the quality evaluations of TCM. To evaluate the quality of ZRR, some scholars have measured various components. For example, Zhang determined the contents of 6 kinds of gingerols (6-gingerol, 8-gingerol, 10-gingerol, 6-shogaol, 8-shogaol, and 10-shogaol) and 4 kinds of curcumins (tetrahydrocurcumin, curcumin, demethoxycurcumin, and bis-demethoxycurcumin) [97]. Meng determined the content of gingerols by reverse high-performance liquid chromatography [98]. To evaluate the chemical constituents of ZRR more comprehensively, HPLC and UPLC fingerprinting was established for quality control of ZRR [99, 100]. In addition, fingerprinting was also used to analyze the quality of ZRR from different regions [100] and the composition and content changes during processing [101]. There are many volatile components in ZRR, and some scholars have established GC fingerprints of volatile components in ZRR and claimed that these methods can be used to evaluate the quality of ZRR [102, 103]. Yet no quality assessment methods associated with the pharmacological activity of ZRR have been devised. It is obviously inadequate to evaluate the quality of ZRR based on chemical constituents alone, warranting the establishment of more activity-related or chemical-activity-based quality evaluation methods.

## 8. Concluding and Perspectives

ZRR is widely used as a medicine and food homologous variety, especially in tropical and subtropical regions. In traditional medicine, many prescriptions containing ZRR are still in use, such as Shengjiang Xiexin Tang and Guizhi Tang. Many clinical practices have proved that the application of ZRR in preventing vomiting is successful. Modern pharmacological research has gradually verified its antiemetic effect and discussed the anticancer, anti-inflammatory, and antioxidation effects. Extracts and isolates from ZRR have multiple medical functions, especially for the nervous system and the cardiovascular and cerebrovascular system. However, there are still some problems that need further study and discussion.

First of all, a variety of phytochemicals have been isolated from this plant. Gingerols are considered to be the main bioactive components, especially 6-gingerol. We have been reported many biological activities of 6-gingerol, and other components such as 6-shagaol have also been reported to have significant pharmacological activities, which deserves more attention.

Secondly, most pharmacological studies have only used a simple animal model, without further study of potential mechanisms of action, such as involved receptors or intercellular and intracellular pathways. In addition, the results of some methods used in animal research are not reliable due to the lack of the positive control group; in some studies, the lack of dose effect analysis has important limitations, and the application of dose needs further research to determine the optimal effective and safe dosage.

Thirdly, although ZRR possesses a variety of potential therapeutic effects, such as antioxidant, anti-inflammatory, neuroprotective, and cardio cerebrovascular protection, these studies have only been carried out in animal and cell models, and few clinical studies have been conducted in humans. As there is a long way to go from the test bed to the bedside, there is no acceptable evidence for clinical use of the plant. Future studies should focus on the biological activity of ZRR in human clinical studies. These studies will contribute to the future medical application of ZRR.

Fourth, the current quality control of ZRR is mainly based on the content requirements of gingerols. For example, 6-gingerol, 8-gingerol, and 10-gingerol are selected as the quality control indicators of ZRR in the Chinese Pharmacopoeia, and the minimum content is made. However, the content of ingredients cannot scientifically explain the quality of ZRR. The quality evaluation of ZRR should be related to clinical efficacy. The quality standard research of ZRR in the future is not only related to the content of components, but also to the pharmacological activity. In order to better define the quality of ZRR, the quality markers of pharmacodynamics are selected as the measurement indexes of quality standards. In recent years, the fingerprint analysis of traditional Chinese medicine is becoming more and more important in the analysis of traditional Chinese medicine. Future research can better define the content and change of components with the help of traditional Chinese medicine fingerprint, so as to ensure the stability of the quality of ZRR.

In conclusion, the botanical characterizations, traditional usages, chemical components, pharmacological activities, toxicity, and quality control of Zingiberis Rhizoma Recens were reviewed in this paper. In recent years, with the development of science and technology, ZRR has made significant progress in chemical components and pharmacological activities, but there are still some challenges. How to clarify the mechanism of traditional uses and modern pharmacological activities and how to establish the correlation research between chemical components and pharmacological activities are the focus of research. It is anticipated that this study can provide a basis for further study of ZRR and promote the long-term development.

## Figures and Tables

**Table 1:** Traditional and clinical uses of ZRR in China.

Preparation name	Main compositions	Formulation	Traditional and clinical uses	Ref.
Shengjiang Xiexin Tang	Zingiberis Rhizoma Recens, Glycyrrhizae Radix Et Rhizoma Praeparata Cum Melle, Ginseng Radix Et Rhizoma, Zingiberis Rhizoma, Scutellariae Radix, Pinelliae Rhizoma, Coptidis Rhizoma, Jujubae Fructus.	Decoction	Epigastralgia, gastroptosis, gastrectasis, chronic gastritis, and other diseases.	[4]
Guizhi Shengjiang Zhishi Tang	Cinnamomi Ramulus, Zingiberis Rhizoma Recens, Aurantii Fructus Immaturus.	Decoction	Chronic gastritis, gastroptosis, or coronary heart disease and angina pectoris caused by chest stuffiness and cardialgia, phlegm retention, and deficiency of Yang Qi in the heart and stomach.	[5]
Guizhi Tang	Cinnamomi Ramulus, Paeoniae Radix Alba, Zingiberis Rhizoma Recens, Jujubae Fructus, Glycyrrhizae Radix Et Rhizoma Praeparata Cum Melle	Decoction	Common cold, influenza, unexplained low fever, and deficiency syndrome of exogenous wind and cold.	[4]
Houpo Shengjiang Gancao Banxia Renshen Tang	Magnoliae Officinalis Cortex, Zingiberis Rhizoma Recens, Pinelliae Rhizoma, Glycyrrhizae Radix Et Rhizoma Praeparata Cum Melle, Ginseng Radix Et Rhizoma.	Decoction	Gastric ulcer and abdominal distension.	[4]
Wuzhuyu Tang	Euodiae Fructus, Zingiberis Rhizoma Recens, Ginseng Radix Et Rhizoma, Jujubae Fructus.	Decoction	Chronic gastritis, vomiting during pregnancy, neurogenic vomiting, nervous headache, and aural vertigo.	[4]
Zhen Wu Tang	Poria, Paeoniae Radix Alba, Zingiberis Rhizoma Recens, Typhonii Rhizoma, Atractylodis Macrocephalae Rhizoma.	Decoction	Chronic glomerulonephritis, cardiogenic edema, hypothyroidism, chronic bronchitis, chronic enteritis, and intestinal tuberculosis.	[4]

**Table 2:** Gingerols isolated from *Z. officinale*.

No.		Chemical component	Formula	Ref.
	Gingerols			
1		4-Gingerol	C_15_H_22_O_4_	[9]
2		Methyl-4-gingerol	C_16_H_24_O_4_	[9]
3		5-Gingerol	C_16_H_24_O_4_	[9]
4		6-Gingerol	C_17_H_26_O_4_	[10]
5		Methyl-6-gingerol	C_18_H_28_O_4_	[10]
6		8-Gingerol	C_19_H_30_O_4_	[10]
7		10-Gingerol	C_21_H_34_O_4_	[10]
8		12-Gingerol	C_23_H_38_O_4_	[11]
9		Acetoxy-4-gingerol	C_17_H_24_O_5_	[12]
10		Acetoxy-6-gingerol	C_19_H_28_O_5_	[12]
11		Acetoxy-8-gingerol	C_21_H_32_O_5_	[10]
12		Acetoxy-10-gingerol	C_23_H_36_O_5_	[10]

	Shogaols			
13		4-Shogaol	C_15_H_20_O_3_	[10]
14		Methyl-4-shogaol	C_16_H_22_O_3_	[9]
15		5-Shogaol	C_16_H_22_O_3_	[9]
16		6-Shogaol	C_17_H_24_O_3_	[10]
17		Methyl-6-shogaol	C_18_H_26_O_3_	[9]
18		8-Shogaol	C_19_H_28_O_3_	[10]
19		Methyl-8-shogaol	C_20_H_30_O_3_	[9]
20		10-Shogaol	C_21_H_32_O_3_	[10]
21		12-Shogaol	C_23_H_36_O_3_	[10]

	Paradols			
22		6-Paradol	C_17_H_26_O_3_	[10]
23		Methyl-6-paradol	C_18_H_28_O_3_	[9]
24		8-Paradol	C_19_H_30_O_3_	[13]
25		Methyl-8-paradol	C_20_H_32_O_3_	[9]
26		10-Paradol	C_21_H_34_O_3_	[12]
27	Zingerone		C_11_H_14_O_3_	[13]

	Gingerdione			
28		6-Gingerdione	C_17_H_24_O_4_	[12]
29		8-Gingerdione	C_19_H_28_O_4_	[13]
30		10-Gingerdione	C_21_H_32_O_4_	[10]
31		12-Gingerdione	C_23_H_36_O_4_	[9]
32		1-Dehydro-6-gingerdione	C_17_H_22_O_4_	[13]
33		1-Dehydro-8-gingerdione	C_19_H_26_O_4_	[13]
34		1-Dehydro-10-gingerdione	C_21_H_30_O_4_	[13]
35		1-Dehydro-12-gingerdione	C_23_H_34_O_4_	[13]

	Gingerdiol			
36		4-Gingerdiol	C_15_H_24_O_4_	[9]
37		6-Gingerdiol	C_17_H_28_O_4_	[10]
38		8-Gingerdiol	C_19_H_32_O_4_	[9]
39		10-Gingerdiol	C_21_H_36_O_4_	[9]
40		5-Acetoxy-4-gingerdiol	C_17_H_26_O_5_	[9]
41		Methyl-5-acetoxy-4-gingerdiol	C_18_H_28_O_5_	[9]
42		5-Acetoxy-6-gingerdiol	C_19_H_30_O_5_	[10]
43		Methyl-5-acetoxy-6-gingerdiol	C_20_H_32_O_5_	[10]
44		Diacetoxy-4-gingerdiol	C_19_H_28_O_6_	[10]
45		Methyl-diacetoxy-4-gingerdiol	C_20_H_30_O_6_	[9]
46		Diacetoxy-6-gingerdiol	C_21_H_32_O_6_	[12]
47		Methyl-diacetoxy-6-gingerdiol	C_22_H_34_O_6_	[12]
48		Diacetoxy-8-gingerdiol	C_23_H_36_O_6_	[9]
49		Methyl-diacetoxy-8-gingerdiol	C_24_H_38_O_6_	[9]

**Table 3:** Essential oils isolated from *Z. officinale*.

No.		Chemical component	Formula	Ref.
	Monoterpenoids			
50		*α*-Pinene	C_10_H_16_	[14]
51		Camphene	C_10_H_16_	[14]
52		*β*-Myrcene	C_10_H_16_	[15]
53		*β*-Phellandrene	C_15_H_16_	[14]
54		1, 8-Cineole	C_10_H_18_O	[14]
55		p-Cymene	C_10_H_14_	[15]
56		*α*-Terpinene	C_10_H_16_	[16]
57		Geraniol	C_10_H_18_O	[14]
58		*α*-Terpineol	C_10_H_18_O	[14]
59		Tricyclene	C_10_H_16_	[14]
60		*β*-Pinene	C_10_H_16_	[14]
61		*α*-Phellandrene	C_15_H_16_	[14]
62		Sabinene	C_10_H_16_	[14]
63		Citronellal	C_10_H_18_O	[14]
64		Terpinolene	C_10_H_16_	[14]
65		Bornyl acetate	C_12_H_20_O_2_	[15]
66		Nerol	C_10_H_18_O	[16]
67		Linalool	C_10_H_18_O	[14]
68		Camphor	C_10_H_16_O	[15]
69		Borneol	C_10_H_18_O	[14]
70		Citronellol	C_10_H_20_O	[14]
71		Geranial	C_10_H_16_O	[17]
72		Geranic acid	C_10_H_16_O_2_	[17]
73		Citronellyl acetate	C_12_H_22_O_2_	[14]
74		Geranyl acetate	C_12_H_20_O_2_	[14]

	Sesquiterpenoids			
75		*α*-Zingiberene	C_15_H_24_	[14]
76		*α*-Curcumene	C_15_H_22_	[14]
77		*β*-Bisabolene	C_15_H_24_	[15]
78		*β*-Sesquiphellandrene	C_15_H_24_	[14]
79		Nerolidol	C_15_H_26_O	[15]
80		*β*-Farnesene	C_15_H_24_	[15]
81		*α*-Bergamotene	C_15_H_24_	[16]
82		Germacrene D	C_15_H_24_	[15]
83		Germacrene B	C_15_H_24_	[14]
84		*α*-Bisabolol	C_15_H_26_O	[14]
85		*α*-Bisabolene	C_15_H_24_	[14]
86		*β*-Eudesmol	C_15_H_26_O	[16]
87		Cyclosativene	C_15_H_24_	[14]
88		*β*-Elemol	C_15_H_26_O	[14]
89		*β*-Elemene	C_15_H_24_	[14]
90		*δ*-Elemene	C_15_H_24_	[16]
91		*γ*-Elemene	C_15_H_24_	[16]
92		*γ*-Eudesmol	C_10_H_18_O	[14]
93		*α*-Eudesmol	C_10_H_18_O	[15]
94		Thujopsene	C_15_H_24_	[16]

**Table 4:** Diarylheptanoid compounds isolated from *Z. officinale*.

No.	Chemical component	Ref.
95	5-Hydroxy-1-(3, 4-dihydroxy-5-methoxyphenyl)-7-(4-hydroxy-3-methoxyphenyl)-3-heptanone	[13]
96	3-Acetoxy-5-hydroxy-1-(4-hydroxy-3-methoxyphenyl)-7-(3, 4-dihydroxyphenyl) heptanes	[13]
97	5-Hydroxy-l, 7-bis (4-hydroxy-3-methoxyphenyl)-3-heptanone	[13]
98	5-Hydroxy-1-(4-dihydroxy-3-methoxyphenyl)-7-(3, 4-dihydroxyphenyl)-3-heptanone	[13]
99	3, 5-Diacetoxy-7-(4-dihydroxy-3-methoxyphenyl)-1-(3, 4-dihydroxy-5-methoxyphenyl) heptane	[13]
100	(3S, 5S)-3, 5-Diacetoxy-1, 7-bis(4-hydroxy-3-methoxy-phenyl) heptane	[18]
101	(3R, 5S)-3-Acetoxy-5-hydroxy-1, 7-bis(4-hydroxy-3-methoxyphenyl) heptane	[18]
102	(3R, 5S)-3, 5-Dihydroxy-1-(4-hydroxy-3, 5-dimethoxyphenyl)-7-(4-hydroxy-3-methoxyphenyl) heptane	[18]
103	(3R, 5S)-3, 5-Diacetoxy-1,7-bis (4-hydroxy-3-methoxyphenyl) heptane	[18]
104	(3R, 5S)-3, 5-Diacetoxy-1-(4-hydroxy-3, 5-dimethoxyphenyl)-7-(4-hydroxy-3-methoxy-phenyl) heptane	[18]
105	(3S, 5S)-3, 5-Dihydroxy-1, 7-bis (4-hydroxy-3-methoxyphenyl) heptane	[18]
106	(3R,5S)-3,5-Dihydroxy-1,7-bis (4-hydroxy-3-methoxyphenyl) heptane	[18]
107	(3S, 5S)-3, 5-Dihydroxy-1-(4-hydroxy-3, 5-dimethoxyphenyl)-7-(4-hydroxy-3-methoxyphenyl) heptane	[18]
108	(5S)-5-Acetoxy-1, 7-bis (4-hydroxy-3-methoxyphenyl)heptan-3-one	[18]
109	5-Hydroxy-1-(3, 4-dihydroxy-5-methoxyphenyl)-7-(4-hydroxy-3-methoxyphenyl) heptan-3-one	[18]
110	5-Hydroxy-1-(4-hydroxy-3-methoxyphenyl)-7-(3, 4-dihydroxy-5-methoxyphenyl) heptan-3-one	[18]
111	5-Hydroxy-1-(4-hydroxy-3-methoxyphenyl)-7-(3, 4-dihydroxyphenyl)heptan-3-one	[18]
112	1, 5-Epoxy-3-hydroxy-1-(4-hydroxy-3, 5-dimethoxyphenyl)-7-(4-hydroxy-3-methoxyphenyl) heptane	[18]
113	3-Acetoxy-1, 5-epoxy-1-(3, 4-dihydroxy-5-methoxyphenyl)-7-(4-hydroxy-3-methoxy-phenyl) heptane	[18]
114	1, 7-Bis (4-hydroxy-3-methoxyphenyl) hept-4-en-3-one	[18]
115	1, 5-Epoxy-3S-hydroxy-1-(3, 4-dihydroxy-5-methoxyphenyl)-7-(4-hydroxy-3-methoxyphenyl) heptane	[19]
116	1, 5-Epoxy-3R-hydroxy-1-(3, 4-dihydroxy-5-methoxyphenyl)-7-(4-hydroxy-3-methoxyphenyl) heptane	[19]
117	3, 5-Diacetyl-1-(3-methoxy-4, 5-dihydroxyphenyl)-7-(4-hydroxy-3-methoxyphenyl) heptane	[19]
118	3-Acetoxy-l, 5-epoxy-l-(3, 4-dihydroxy-5-methoxyphenyl)-7-(4-hydroxy-3-methoxyphenyl) heptane	[20]

**Table 5:** Pharmacological action of ZE or certain ingredients.

Pharmacological activity	Assay	Subject	Treatment	Effect	Ref.
Treatment of AD	The Morris water maze test	*Aβ*_25−35_ and aluminum chloride induced memory impairment rat model	ZAE (1, 2, and 4 g/kg)	The latency of water maze test shortened.	[25]
Improving cognition	Novel object recognition test	A mice model of scopolamine-induced memory deficits	ZSE (5, 25, and 125 mg/kg)	ZE induced the upregulation of NGF and increased the levels of ERK and CREB in the hippocampus.	[26]
Cardioprotective activity	Cardioprotective activity evaluation test	Rat ventricular myocytes	6-Gingerol (3, 10, 30, 100, and 300 *μ*mol/L)	6-Gingerol reduced the L-type Ca^2+^ in a concentration-dependent manner with a half-maximal inhibitory concentration (IC_50_) of 31.25 *μ*mol/L in cardiomyocytes.	[27]
Cerebral ischaemia protection	Cerebral ischemia-reperfusion assay	Rats	ZAE (200, 100, and 50 mg/kg)	ZAE decreased the contents of Glu, Ca^2+^ and water in brain tissue and increased the activities of SOD.	[28]
Antiemetic effect	Intestinal tension test	Isolated guinea pig ileum	6-Gingerol and 6-shogaol (3 × 10^−5^,1 × 10^−5^,3 × 10^−6^ mol/L)	They dose-dependently inhibited the increase of contraction of the guinea pig ileum stimulated by 5-HT and 2-me-5-HT, reduced the mean tension.	[29]
Antimicrobial effect	The KB disc diffusion method	*Aspergillus flavus* and *Aspergillus niger*	ZEE	The antibacterial diameter of ZEE to *Aspergillus niger* was 21.80 mm and to *Aspergillus flavus* was 16.83 mm.	[30]
Antitumour effect	MTT cell proliferation assay; comet assay (single‐cell gel electrophoresis)	Human colon cancer cell line, HCT116	Zingerone (ZO) (2.5, 5, and 10 *µ*M)	ZO drastically reduced cell proliferation and increased the percentage of DNA damage in HCT116 cells.	[31]
Anti-inflammatory effect	In vivo anti-inflammatory effect test	5-week-old female mice	ZEE (100, 300, and 500 mg/kg)	Body weight, colon length, and DAI values of mice improved.	[32]
Anti-oxidative effect	DPPH and ferric reducing antioxidant properties (FRAP) assay	Albino rats	ZME (2.5–100 *μ*g/mL)	The extract promoted an inhibition of free radicals with IC_50_ values of 47.05 ± 2.03 *μ*g/mL and 89.15 ± 0.29 *μ*g/mL in DPPH and FRAP assay.	[33]

**Table 6:** Toxicological studies of ZRR.

Assay	Subject	Treatment	Effect	Ref.
Oral toxicity test	Mice	ZAE and ZME (2, 4, 6, 8, and 10 g/kg)	No toxic effects were observed at a higher dose of 10 g/kg body weight.	[89]
Maximum tolerance test	Male and female mice	ZAE and ZEE	The maximum tolerance of ZE on male and female mice was greater than 66.67 g/kg.	[93]
Acute toxicity test (a 14-day study)	Healthy Sprague-Dawley rats	ZEE (5000 mg/kg)	No evidence of toxicity and death was observed in all rats.	[94]
Subacute toxicity test (a 28-day study)		ZEE (500, 1000, and 2000 mg/kg)	At different doses, only significant differences in hematological and biochemistry parameters were observed.	
Chronic toxicity test (a 12-month study)		ZEE (250, 500, and 1000 mg/kg)		
Cell viability assay	All cell lines of hepatoma (HepG2), breast carcinoma (MDA-MB-231), and lung adenocarcinoma (A549)	ZSE (20–100 *μ*m)	Compared with the control cells, the cell viability of all cancer cells was significantly lost.	[16]
Bone marrow chromosomal aberration test (a 7-day study)	Male Wistar rats	ZAE (100 mg/kg, 200 mg/kg)	The chromosome structure of bone marrow cells was not degraded.	[95]
Micronuclei tests			ZAE showed a more structured layout and healthy polychromatic erythrocyte.	
Reproductive toxicity test (a 28-day study)	Adult male Sprague-Dawley rats	ZEE (1 g/kg)	Hormone levels, trace elements, and antioxidant enzyme activity increased, while the contents of total homocysteine and malondialdehyde decreased.	[96]

**Table 7:** The quality control of *Z. officinale* in Pharmacopoeia of different countries.

Name	Medicinal part	Pharmacopeia	Water detection or loss on drying	Ash detection		Volatile oil content	Extract content	Determination of content	
				Total ash	Acid insoluble ash			Marker components	Content requirements
Zingiberis Rhizoma Recens	Fresh rhizome	Chinese Pharmacopeia (vision 2015)	—	≤2.0%	—	≥0.12% (ml/g)	—	6-Gingerol;	≥0.05%;
								8-Gingerol and 10-gingerol	The total content ≥0.04%
Zingiberis Rhizoma	Dried rhizome	Chinese Pharmacopeia (vision 2015)	≤19.0%	≤6.0%	—	≥0.8% (ml/g)	≥22.0%	6-Gingerol	≥0.6%
Zingiberis Rhizoma	Rhizome	Japanese Pharmacopeia (vision 2017)	—	≤8.0%	—	—	—	6-Gingerol	≥0.3%
Zingiberis Processum Rhizoma	Rhizome	Japanese Pharmacopeia (vision 2017)	≤15.0%	≤6.5%	≤1.5%	—	≥8.0%	—	—
Zingiberis Rhizoma	Dried rhizome	United States Pharmacopeial Convention (vision 2017)	≤10.0%	≤8.0%	≤2.0%	≥1.8 ml/100 g	≥10.0%	Gingerols and gingerdiones; shogaols	The total content ≥0.8% ≤ 0.18%
Zingiberis Rhizoma	Dried rhizome	European pharmacopoeia (vision 8.0)	≤100 ml/kg	≤6.0%	-	≥15 ml/kg	—	—	—
Zingiberis Rhizoma	Dried rhizome	Korean Pharmacopoeia (KP X)	—	≤8.0%	—	—	—	6-Gingerol	≥0.4%
Sunthi	Dried rhizome	Indian Pharmacopoeia (vision 2010)	≤12.0%	≤8.0%	≤1.5%	—	≥2.0%	Total gingerols	≥0.8%

Cited from the Drug Standard Database at https://www.drugfuture.com/standard/search.aspx.

## Abbreviations

*Z. officinale*: *Zingiber officinale* Roscoe
ZRR: Zingiberis Rhizoma Recens
ZAE: Aqueous extract of Zingiberis Rhizoma Recens
ZEE: Ethanolic extract of Zingiberis Rhizoma Recens
ZSE: Supercritical extract of Zingiberis Rhizoma Recens
ZME: Methanol extract of Zingiberis Rhizoma Recens
AD: Alzheimer's disease
CysLT1R: Cysteinyl leukotriene 1 receptor
A*β:*: Amyloid beta
Akt: Protein kinase
GSK-3*β*: Glycogen synthase kinase-3*β*
PI3K: Phosphoinositide-3 kinase
PC cells: Pheochromocytoma cells
NGF: Nerve growth factor
ERK: Extracellular-signal-regulated kinase
CREB: Cyclic AMP response element-binding protein
LPS: Lipopolysaccharide
ROS: Reactive oxygen species
BNIP3: Bcl-2 E1B 19-KDa interacting protein 3
TNF-*α*: Tumor necrosis factor-*α*
CK: Creatine kinase
LDH: Lactate dehydrogenase
FCI-R: Focal cerebral ischemia-reperfusion
MCAO: Middle cerebral artery occlusion
Caspase: Cysteinyl aspartate specific proteinase
Bax: Bcl-2 associated *X* protein
Bcl-2: B-cell lymphoma/leukemia-2
CI-R: Cerebral ischemia-reperfusion
EAA: Excitatory amino acid
ICAM-1: Intercellular adhesion molecule-1
P-selectin: Platelet-selectin
SOD: Superoxide dismutase
MDA: Malondialdehyde
5-HT: 5-Hydroxytryptamine
NK: Neurokinin
CINV: Chemotherapy-induced nausea and vomiting
PONV: Postoperative nausea and vomiting
MIC: Minimum inhibitory concentration
STAT-3: Signal transducer and activating transcription-3
PARP-1: Poly ADP ribose polymerase 1
PGE2: Prostaglandin E2
NF-*κ*B: Nuclear factor-kappa B
COX-2: Cyclooxygenase-2
INOs: Inducible nitric oxide synthases
IL-1*β*: Interleukin-1*β*
IL-6: Interleukin-6
IL-10: Interleukin-10
MAPK: Mitogen-activated protein kinase
DAI: Disease activity index
Nrf2: Nuclear factor erythroid 2-related factor 2
HO-1: Hemeoxygenase-1
DPPH: 1, 1-Diphenyl-2-picrylhydrazyl.

## Additional Points

We followed the methods of Hanyan Luo et al., 2019 [104].
